# Multifocal nodular steatosis mimicking liver metastasis

**DOI:** 10.1590/S1679-45082017AI3869

**Published:** 2017

**Authors:** Eduardo Kaiser Ururahy Nunes Fonseca, Thiago Raspa Freitas Magdalena, Fernando Ide Yamauchi, Marcelo de Castro Jorge Racy, Cássia Franco Tridente, Ronaldo Hueb Baroni

**Affiliations:** 1 Hospital Israelita Albert Einstein, São Paulo SP, Brazil.

We report a case of a 56-year-old male under investigation for large lytic lesion at clivus. After lesion resection, patient was referred to abdominal computed tomography scan for metastasis screening. The lesion was posteriorly characterized as chondroid chordoma at histopathology examination.

On non-enhanced computed tomography, multiple hypottenuating nodules of different sizes were seen; the largest nodule had 3.2cm. On post-contrast phases lesions had similar enhancement to adjacent liver parenchyma. However, no significant mass effect or invasiveness were seen, once vascular structures (liver veins and portal branches) crossed such nodules without dislocation or invasion. Our findings, although not pathognomonic, suggested the hypothesis of multifocal nodular steatosis.

In order to confim the diagnosis, patient underwent a magnetic resonance imaging (MRI) that further characterized lipid within the lesions. On MRI this characteristic can be explored by chemical shift technique in which there is signal drop on out-of-phase sequence compared to in-phase sequence.

Fat deposits in liver parenchyma are frequent and have a prevalence of 15% in general population.^(1,2)^ Nodular patterns are uncommon, and can be mistaken for metastasis, which is particularly problematic in oncologic patients. In our case, patient had a chordoma, a rare tumor that can present with metastasis in 3 to 48% of cases – and about one fifth of them are located in the liver. ^(3)^

Imaging findings such as lack of invasion or displacement of vascular structures, enhancement similar to normal liver parenchyma and stability over time corroborate the diagnosis. Magnetic resonance imaging is very helpful to confirm this diagnosis with signal drop on out-of-phase sequence compared to in-phase sequence, confirming intracellular lipid content.^(2,4-7)^ [Fig f01] [Fig f02] [Fig f03]

**Figure 1 f01:**
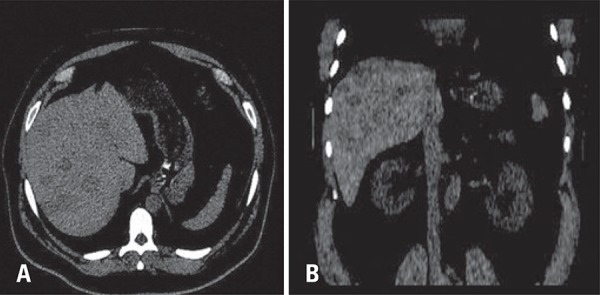
(A) Non-enhanced computed tomography on axial A coronal reformatted. (B) Showing multiple hypoattenuating liver nodules

**Figure 2 f02:**
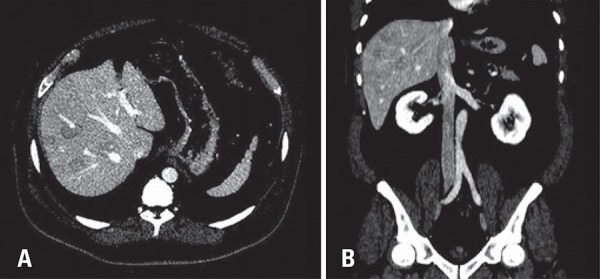
(A) Post-contrast computed tomography on axial A and coronal reformatted. (B) Showing multiple hypoattenuating liver nodules. Notice vascular structures crossing inside the nodules without deviations indicating lack of mass effect

**Figure 3 f03:**
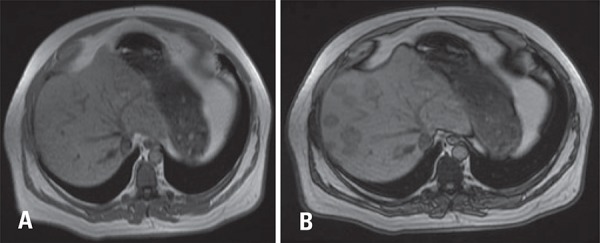
Magnetic resonance imaging in gradient echo sequences. (A) In-phase and out-of-phase images. (B) Show signal drop on the latter, indicating intracellular lipid content
